# Accumulation of Arsenic by Plants Growing in the Sites Strongly Contaminated by Historical Mining in the Sudetes Region of Poland

**DOI:** 10.3390/ijerph17093342

**Published:** 2020-05-11

**Authors:** Agnieszka Dradrach, Anna Karczewska, Katarzyna Szopka, Karolina Lewińska

**Affiliations:** 1 Institute of Agroecology and Plant Production, Wrocław University of Environmental and Life Sciences, pl. Grunwaldzki 24a, 50-350 Wrocław, Poland; agnieszka.dradrach@upwr.edu.pl; 2 Institute of Soil Science and Environmental Protection, Wrocław University of Environmental and Life Sciences, ul. Grunwaldzka 53, 50-357 Wrocław, Poland; katarzyna.szopka@upwr.edu.pl; 3 Department of Soil Science and Remote Sensing of Soils, Adam Mickiewicz University in Poznań, ul. Krygowskiego 10, 61-680 Poznań, Poland; karolina.lewinska@amu.edu.pl

**Keywords:** arsenic, accumulation, extractability, translocation factor, bioaccumulation factor, bioconcentration factor

## Abstract

The uptake of As by various plants growing in highly enriched sites was examined in order to identify potential As accumulators and to assess the risk associated with As presence in plant shoots. Representative samples of 13 plant species, together with soil samples, were collected from various sites affected by historical As mining: mine and slag dumps, tailings and contaminated soils with As concentrations in a range 72–193,000 mg/kg. Potentially and actually soluble As forms, extracted with 0.43 M HNO_3_ and, 1M NH_4_NO_3_ were examined in relation to As concentrations in plant roots and shoots. The latter differed strongly among the species and within them and were in the ranges 2.3–9400 mg/kg and 0.5–509 mg/kg, respectively. The majority (over 66%) of plant samples had As shoot concentrations above 4 mg/kg, an upper safe limit for animal fodder. The uptake of As by plants correlated well with total and extractable soil As, though As concentrations in plants could not be predicted based on soil parameters. *Equisetum* spp. and *C. epigejos* indicated a particularly strong accumulation of As in shoots, while *A. capillaris*, and *H. lanatus* showed a limited As root-to-shoot transfer, apparently associated with species-related tolerance to As.

## 1. Introduction

Arsenic is a naturally occurring soil component that due to anthropogenic activities can be locally concentrated to the levels that negatively affect human health and functioning of ecosystems. If present in high concentrations, it can pose a particular risk to humans and animals because of its toxicity, mutagenicity and carcinogenicity [1,2,3]. Numerous papers have focused on the issues of drinking water pollution in black foot disease endemic areas [4,5,6], as well as on the accumulation of As by rice from soils irrigated by As-contaminated water [7,8,9,10]. Arsenic can also cause various disturbances in ecosystems, however, the ecological effects of its presence in soils in high amounts were less extensively examined [11]. Obviously, enhanced uptake of As in plants can pose a considerable risk to wild and domestic consumers by entering a food chain. 

Arsenic is usually poorly soluble in soils and is poorly taken up by plants [12,13,14,15]. The aboveground parts of plants usually contain small concentrations of As, below 1 mg/kg [16,17]. The permissible level of As in dry fodder has been established at a level of 4 mg/kg [18,19]. It has been proved that several plant species growing in strongly contaminated sites can develop various tolerance mechanisms that lead to reduced uptake of As, either by adaptation of the arsenate uptake system or by avoidance and exclusion [20,21,22,23,24,25]. Mycorrhiza, as well as rhizospheric and endophytic bacteria can additionally reduce the influx of As to plant roots therefore supporting the tolerance to its high concentrations in soils [26,27,28]. On the other hand, however, some plants species, particularly those growing in aquatic conditions or wetlands, can intensively take up As and tolerate its high concentrations in shoots. An extreme case of that phenomenon is As-hyperaccumulating species [29,30], though, their natural occurrence was not reported from Europe. However, there are several reports on strong accumulation of As in plants growing in strongly polluted sites, in particular in those affected by mining activity, so that its concentrations in plant shoot tissues can be as high as tens, hundreds or even thousands mg/kg [15,31,32,33,34,35,36,37,38,39]. 

There are three historical As mining sites in the Sudetes (SW Poland) where As ores were exploited and processed over centuries: Złoty Stok (former German name: Reichenstein, N 50°26–28’, E 16°51–54’), Radzimowice (N 50°56’27–44’’, E 15°57–58’) and Czarnów (N 50°48’15–34’’, E 15°54–55’’), of which Złoty Stok, that operated until 1962, was the largest one. The concentrations of As in waste rocks disposed on numerous waste dumps, and those in tailings and soils, are there as high as thousands mg/kg [40,41,42,43,44], while the concentrations of soil As, considered environmentally safe, have been set by Polish law at the levels 10–100 mg/kg, depending on soil properties and land usage [45,46].

It is important to examine the uptake of As by various plant species in such unfavorable conditions, and to recognize the factors governing As translocation to plant shoots, often described by a translocation factor TF (1) [47]. Another biogeochemical index, a bioaccumulation factor BAF, based on total concentrations of As in soils and plant tissues, is commonly used to assess the potential of As uptake from soils (2) [48]. Another index, a bioconcentration factor BCF (defined in analogy to aquatic environments), based the concentrations in soil pore water or on potential or actual solubility/extractability, is sometimes preferred for the assessment of a real risk associated with the presence of toxic elements in soils and its uptake by plants (3) [49,50,51]:

(1) $$\mathrm{TF}=\frac{\mathrm{As}\;\mathrm{in}\;\mathrm{shoots}}{\mathrm{As}\;\mathrm{in}\;\mathrm{roots}}$$

(2) $$\mathrm{BAF}\;\left(\mathrm{shoots}\;\mathrm{or}\;\mathrm{roots}\right)=\frac{\mathrm{As}\;\mathrm{in}\;\mathrm{plant}\;\mathrm{material}\;\left(\mathrm{shoots}\;\mathrm{or}\;\mathrm{roots}\right)}{\mathrm{total}\;\mathrm{As}\;\mathrm{in}\;\mathrm{soil}}$$

(3) $$\mathrm{BCF}\;\left(\mathrm{shoots}\;\mathrm{or}\;\mathrm{roots}\right)=\frac{\mathrm{As}\;\mathrm{in}\;\mathrm{plant}\;\mathrm{material}\;\left(\mathrm{shoots}\;\mathrm{or}\;\mathrm{roots}\right)}{\mathrm{soluble}\;\left(\mathrm{extractable}\right)\;\mathrm{As}\;\mathrm{in}\;\mathrm{soil}}$$

The main aims of this research were: (1) to examine total concentrations of As in plant shoots and roots in the mine activity-affected sites strongly enriched in As in order to assess the risk for potential consumers; (2) to recognize plant species that accumulate relatively high concentrations of As in their shoots and show high values of As bioavailability indices; (3) to check suitability of two ISO-approved extracting solutions for the prediction of As uptake by plants.

## 2. Materials and Methods 

### 2.1. Experimental Areas

The study was carried out in eight areas situated in three historical As mining centres: Złoty Stok, Radzimowice and Czarnów (Table 1). Some of those areas (1,5) were partly barren surfaces of large mine dumps, some others (4,6,7) were situated in forests with numerous smaller mine or slag dumps scattered there throughout, or in grasslands (2,3). Area 6 was additionally affected by acid mine and rock drainage (AMD and ARD). The areas 2 and 7 were typical hay meadows, either occasionally flooded by tailings (2) or affected in the past by ore mining and smelting (7). Area 3 was a dry grassland where soils developed of pure tailings accumulated at the foreland of impoundment. Finally, area 8 represented a mosaic of forested land patches and grasslands with mine dumps of various sizes. In all those areas, soils contained very high concentrations of As [40].

### 2.2. Soil and Plant Sampling

On the basis of a screening study, representative species of grass and herbaceous plants, commonly occurring in all the areas, were chosen for analysis (Table S1). Additionally, two kinds of tree seedlings, that started to inhabit the dumps, were included in the study. In June, i.e. in the time of grass flowering, plant samples were collected from each area in representative 6–8 points.

The plants were sampled, including aboveground parts and roots (or rhizomes), together with about 2 kg lumps of topsoil (0–20 cm). The aboveground parts of plants were separated in the field by harvesting directly above the crowns (or above soil surface), while the soil lumps with underground parts of plants were transported to the laboratory, where the roots were carefully removed from soil. Soil and plant material was then air-dried and prepared for analysis. Material collected from each sampling point was represented by three subsamples, so all the analysis were performed in triplicates. For statistical analysis, aimed to compare As uptake by various plants, only those species were taken into account that were represented by at least four samples (Table S1).

### 2.3. Soil Analysis

Air-dried soil samples were homogenized, ground, and sieved to 2 mm under special safety conditions that involved a vacuum-driven fume hood and personal protection for technicians (protective clothes, masks and glasses). Aliquots of fine soil material (ca. 200 g) were then analyzed. Soil texture was determined by a sieve-and-hydrometer method [59]. Soil pH was measured potentiometrically in a suspension (1:2.5, v:v) with 1M KCl. Organic carbon (Corg) content in soil was analyzed on the CS-MAT 5500 instrument (Strohlein, Kaarst,. Germany). For determination of “pseudototal” As (termed further “total”), soil samples were digested with aqua regia (HNO_3_ + HCl, 1 + 3) in microwave oven, according to ISO 14466. Potentially soluble forms of As were extracted from soil with 0.43 M HNO_3_ (ISO 17402), and actually soluble As species–with 1 M NH_4_NO_3_ (ISO 19730). Additionally, a soluble P, considered phytoavailable, was determined by a lactate/Ca method [60,61], a routine procedure used in Poland, as the concentrations of P in soil pore water can considerably affect As uptake by plants [62,63,64,65]. Concentrations of As and P in digests and extracts were determined by ICP-AES, on an iCAP 7400 system (Thermo Scientific, Waltham, MA, USA), with determination limits 0.002 and 0.02 mg/L, respectively. All reagents and deionized water were of ultra-pure quality, and all the labware used was either new or thoroughly cleaned prior to usage. Validation of analytical methods involved analysis of two solid CRMs, certified for aqua-regia extracted elements (CNS 392 i CRM 027). All the results of control analyses were considered satisfactory, falling in the range 98–104% of As certified values. Due to the lack of a suitable CRMs, the analytical correctness of As determination in HNO_3_ and NH_4_NO_3_ extracts, was verified via standard addition.

### 2.4. Plant Analysis

Oven-dried (60 °C, 24 h) and ground samples of plant material were pretreated with 30% hydrogen peroxide and digested in concentrated HNO_3_, in a microwave system. The digests were diluted with deionized water and filtered through 0.45 μm syringe filters. The concentrations of As in the digests were determined by ICP-AES as in soil digest. Validation of analytical method involved the analysis of plant CRMs: BCR-414 and DC-7349. Additionally, the analytical results obtained with ICP-AES were randomly controlled by ICP-MS 8800 Triple Quad (Agilent, Santa Clara, CA, USA). The differences between the results obtained with these two methods remained below 20%.

### 2.5. The Indices of As Uptake and Root-to-Shoot Transfer 

In order to characterize the uptake of As by plants in relation to soil As concentrations, as well as to assess the rate of its translocation from roots to the aboveground parts, appropriate indices were calculated. A translocation factor TF was determined as the ratio of As concentrations in plant shoots to those in roots [47]. Shoot/root bioaccumulation factor (BAF) was defined as the ratio of As concentrations in plant material to total As concentrations in soil. Shoot and root bioconcentration factors BCF were calculated in relation to soluble As in soil [50,51], i.e. to its fraction extractable with 1M NH_4_NO_3_ or with 0.43 M HNO_3_.

### 2.6. Statistics

Basic descriptive statistics was applied to evaluate the distributions of data sets obtained from chemical analyses. When necessary, the sets of variables were normalized by log 10 transformation prior to further calculations. For each data sets and each plant species, median values and 25% and 75% percentiles were calculated and visualized in the form of box-and-whiskers plots. Extremes and outliers were neglected in those calculations, though, they were presented in the graphs. 

The relationships between log-normalized soil and plant data have been presented in diagrams. produced by the Excel software. Pearson correlation coefficients were calculated to examine the relationships between soil properties and arsenic concentrations in plant samples. Additionally, principal component analysis (PCA), the multivariate statistical technique, was applied both to the whole sets of data, and to the data characterizing particular plant species separately, in order to extract information about the associations and relationships between the variables. All statistical analyses were performed using Excel 2010 (Microsoft, Albuquerque, NM, USA) and Statistica 13 (StatSoft, Tulsa, OK, USA) softwares.

## 3. Results and Discussion

### 3.1. Soil Properties 

Soil properties differed strongly both among the areas and in most cases, also within them (Table 1). Total As content in soils varied in the range: 72–193,000 mg/kg, with the smallest values in the area 7 (hay meadows and pastures in Radzimowice), and extremely high As accumulation in an alluvial soil affected by AMD and ARD in the area 6. Very high As concentrations, above 10,000 mg/kg, were also reported from various mine dumps. 

Obviously, As present in soils, was mainly inherited from primary As-bearing minerals, such as arsenopyrite, loellingite, their various associations [44,55,57], as well as from secondary minerals and non-crystalline components of different susceptibility to weathering and various solubility. Therefore, the shares of potentially soluble As (extractable with HNO_3_) and actually soluble As (extractable with 1M NH_4_NO_3_) in soils differed strongly among the samples falling in the broad ranges 0.3–88.6% and <0.01–0.83% % of total As, respectively, with the median values: 37.1% and 0.04%. Though, in the whole set of study data, both potentially and actually soluble As concentrations were highly significantly (*p* < 0.001) correlated with total soil As (Table 2). They correlated also with the content of soluble (“bioavailable”) P in soils. Relatively poorer (R = 0.449), though still significant at *p* < 0.001, correlation between 1M NH_4_NO_3_-extractable As and soil pH was undoubtedly caused by the fact that the solubility of As tends to increase both at high pH, due to an anionic character of As-bearing ions, and at extremely low pH, due to dissolution of iron oxides [15]. The pH values in our study were spread in a broad range (2.88–7.66), likely including the zones of enhanced solubility both at low and high pH. Moreover, a relationship between a real As solubility vs. pH can be modified in the field by changing soil redox conditions followed by a possible reductive dissolution of iron oxides [15,43,66], which might have not been reflected after soil drying in lab conditions. 

### 3.2. Arsenic Concentrations in Plants

**Figure 1:**
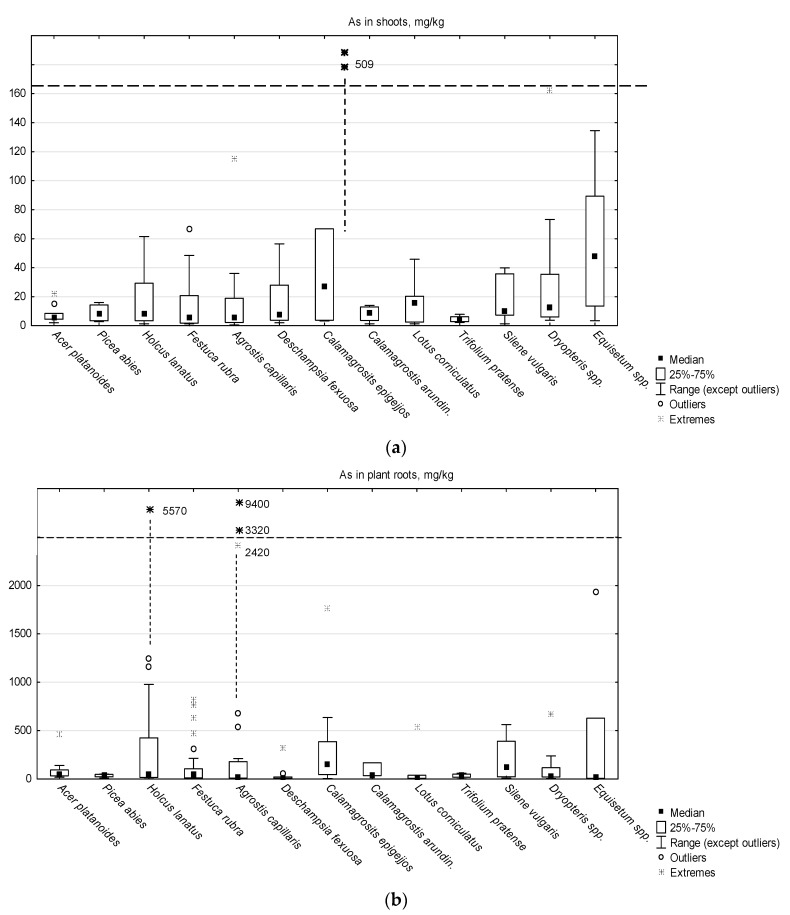
Arsenic concentrations in shoots (**a**) and roots (**b**) of plant species examined.

Box-whiskers plots (Figure 1) show the concentrations of As in shoots and roots of plant species examined. The pictures illustrate a large diversity of plant capability to take up As from soils and to accumulate it in their tissues. Minimum and maximum As concentrations determined in plant samples differed dramatically, more that by three orders of values. However, the median As concentrations, determined separately for particular plant species, listed in Table S2, and indicated in graph as black squares, did not differ so largely.

#### 3.2.1. As in Plant Shoots

Total concentrations of As in the aboveground parts of plants were in a broad range 0.5–509 mg/kg, indicating that the plants growing in our study area can accumulate very high amounts of As in their shoots. The median value, determined for the whole set of results, was 8.9 mg/kg, which was comparable with As concentrations in plant shoots reported by various authors from contaminated environments [15,16,67,68,69,70]. It should be stressed, however, that the majority of results, i.e. 66%, exceeded the value 4 mg/kg, considered a safe concentration of As in animal fodder. In particular, an upper quartile of analytical data should attract special attention, as 25% of all plant samples had the shoot As concentrations above 26.9 mg/kg, and 10% of results exceeded 50.8 mg/kg. Moreover, statistical calculations neglected several outliers and extreme values (i.e. the results > 61 mg/kg) that made up further 8% of particularly high As concentrations. The highest species-related median value of shoot As concentration, 47.5 mg/kg, was determined in *Equisetum* spp., a genus that was already reported as As accumulator [33,40,71]. Its high As accumulation capability can be attributed either to special, genus-related physiological features, not yet more closely examined, or explained by its particular tolerance to temporary soil flooding and As mobilization in reducing conditions.

A relatively high median value of As concentrations in the shoots, 26.8 mg/kg, were also reported for *C. epigejos*, a dominating grass species in the areas 2 and 3. Two extremely high values of As concentrations in plant shoots (478 and 509 mg/kg) were also reported for the representatives of this grass species. They grew in soils with neutral pH (pH > 6.8) and considerably high concentrations of 1M NH_4_NO_3_-extractable As (>2.5 mg/kg). It can be supposed that, unlike three other grass species: *H. lanatus*, *F. rubra* and *A. capillaris*, growing in same conditions (in the areas 1, 2 and 3), the populations of bush grass *C. epigejos* examined in this study did not evolve the mechanisms of tolerance based on As avoidance or reduced influx to the shoots [21,23,72].

#### 3.2.2. As in Plant Roots

As concentrations in plant roots were, in general, much higher and much more differentiated than those in the aboveground parts of plants (Figure 1) and ranged from 2.3 to 9400 mg/kg. Extremely high As concentrations in plant roots (2400 mg/kg and higher), apparently associated with very strong soil enrichment in As, were reported for two grass species: *A. capillaris* and *H. lanatus*, while *C. epigejos* and *S. vulgaris* had the highest median values of root As (126 and 147 mg/kg, respectively). On the contrary, relatively low root As were reported in the case of *D. flexuosa* growing in acidic mine soils, and *L. corniculatus*, that had the median root As: 11.3 and 10.8 mg/kg. 

### 3.3. Translocation Factor TF

**Figure 2:**
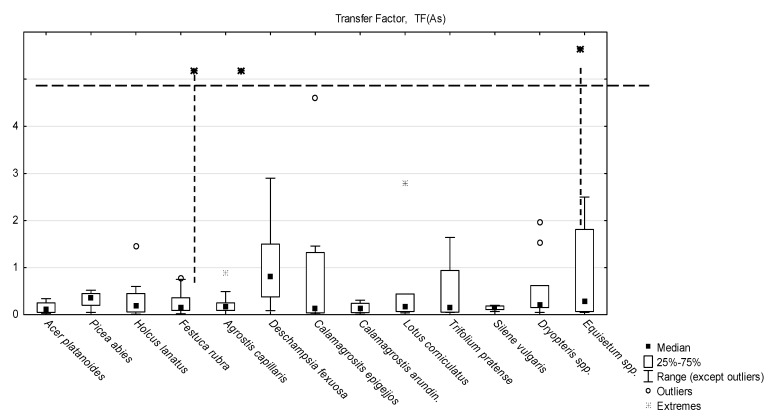
The values of arsenic roots to shoots transfer factor (TF).

Translocation factor TF is a parameter that characterizes plant ability to transfer elements from roots to shoots. The values of TF calculated for all the plants growing in the study areas ranged broadly (0.004–26.8), though, they were generally very low (Figure 2), with a median 0.17, which is a typical feature for As [15,16,39,73,74].

Most of the species-determined median values of TF remained below 0.20. The exceptions were: an unusually high median of 0.82 obtained for *D. flexuosa*, a grass growing in forest habitats in strongly acidic soils (pH 2.88–4.32) on the dumps, as well as the medians for spruce seedlings *P. abies* on acidic mine dump soils: 0.37, the horsetail *Equisetum* spp.: 0.28, and ferns *Dryopteris* spp.: 0.21 (Table S2). It should be stressed, however, that singular exceptionally high values of TF were noted for various species. The TF values above 10 were reported in the cases of *F. rubra, A. capillaris* and *Dryopteris* spp., and they all were associated with strongly acidic soils (pH below 3.8). 

### 3.4. Bioaccumulation Factor BAF

**Figure 3:**
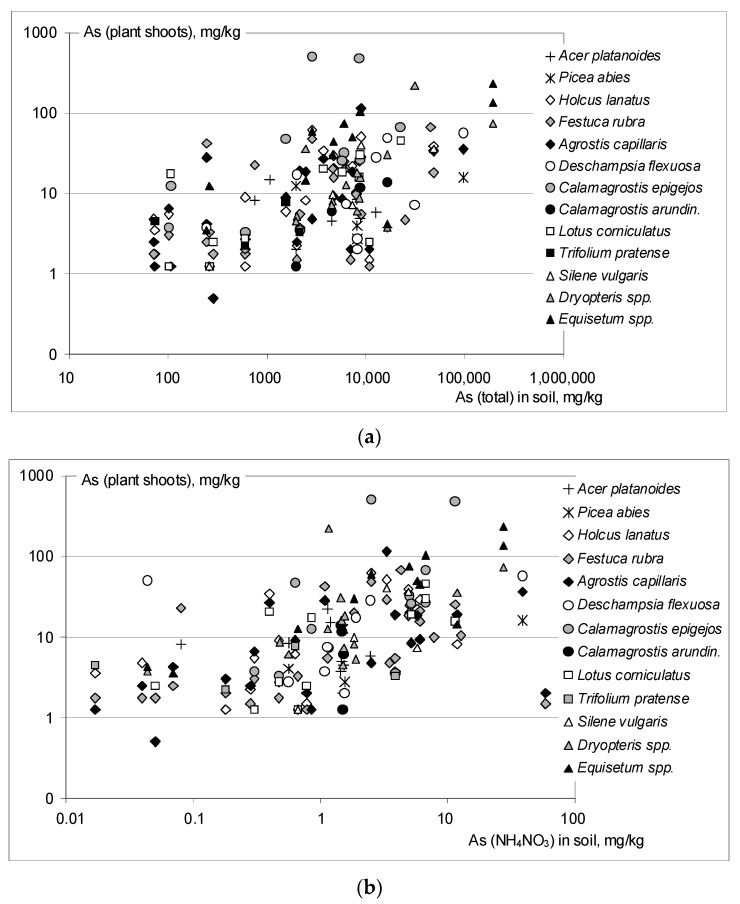
Arsenic concentrations in the aboveground parts of plants, as related to soil As. Graph (**a**) illustrates the relationships between As in the aboveground parts of plants and total soil As, and graph (**b**) relates the concentrations of As in the aboveground parts to 1M NH_4_NO_3_-extractable As in soils.

BAF values, that relate the accumulation of element in plant shoots or roots to its total concentrations in soils, did not differ from those described in similar studies, and were in general very low. The shoot BAF values were in the range <0.001–0.179, with 90% cases below 0.025, and a median value 0.003. The single cases of considerably high BAF coefficients, above 0.1, were noted for grasses: *C. epigejos* (0.179, 0.117), *F. rubra* (0.174) and *A. capillaris* (0.116), as well as for a common bird’s-foot trefoil *L. corniculatus* (0.164) and *Dryopteris* spp. (0.105). The horsetail (*Equisetum* spp.) indicated a relatively high median shoot BAF: 0.008. The relationships between As concentrations in plant shoots and total As in soils are illustrated in the Figure 3. The cases listed above appear as outliers protruding above a cloud of the other data. A clear tendency of increasing As in shoots with increasing soil total As was confirmed by a high correlation coefficient (R = 0.640), significant at *p* < 0.001 (Table 3).

The root BAF values differed strongly within our study, and were obviously higher than the shoot BAFs, falling in the range <0.001–1.13, with a median 0.020. An extraordinarily high BAF value (>1.0) referred to *C. epigejos* growing in soil with slightly acidic pH (4.82). The latter result was checked twice and should be considered as a case of unusually strong capability of that particular bush grass genotype to accumulate As in its roots. No similar cases of such a strong As accumulation in the roots of this grass species are in fact reported in the literature. The median values of root BAF, determined for single plant species separately, were in the range 0.002–0.062, with the lowest values (<0.003) obtained for the ferns *Dryopteris* spp. and the grass *D. flexuosa* that grew in acidic soils and poorly accumulated As in roots/rhizomes. On the contrary, three other grass species: *H. lanatus, A. capillaris* and *C. epigejos*, as well as the representative of Loteae family, *L. corniculatus*, had the highest median values of root BAF, above 0.050. All these three grass species were earlier reported to develop the mechanisms of As tolerance, based on either avoidance or exclusion and apparently supported by mycorrhiza and rhizospheric bacteria. Those mechanisms can be responsible for an increased accumulation of As in plant roots [23,26,28]. The highest values of root BAF were associated with soils highly enriched in As, with its total concentrations in the range 103–104 mg/kg. 

### 3.5. As Extractability and Plant Uptake. Bioconcentration Factors BCF

Considering a limited As solubility in soils, we presumed that the correlations between As concentrations in plant tissues and actually or potentially soluble soil As in soils should be stronger compared to those with total soil As. Correlation coefficients between 1M NH_4_NO_3_-extractable As in soils and As concentrations in plant roots and shoots, determined for the whole collection of samples, were indeed relatively high (R: 0.503 and 0.599, respectively), and highly significant (Table 3). Similarly high and significant were correlations between the root and shoot As and 0.43 M HNO_3_-extractable soil As (R: 0.576 and 0.584). All those R values were, however, lower than corresponding R coefficients calculated for total soil As, and a graphical illustration of plant shoot As vs. soluble soil As (Figure 3) proved a large diversity of experimental data. 

The values of related root and shoot actual bioconcentration factor BCF (calculated in relation to actual As solubility) differed strongly, and were in the ranges <0.1–1210 for shoots and <0.1–8290 for roots, with the medians: 7.4 and 41.5. The BCF values depended on extractable soil As, and decreased with increasing pool of potentially or actually soluble pool of soil As (Table 4). In other words, the higher was the pool of soluble As in soil, the smaller part of this pool was taken up and accumulated by plants.

**Figure 4:**
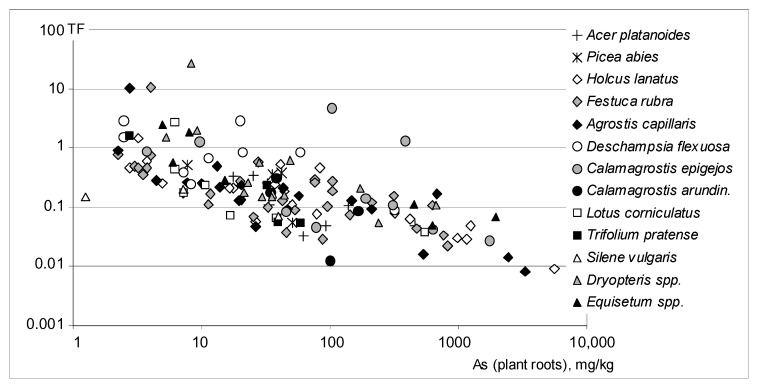
Roots to shoots transfer factor of arsenic (TF) as related to root As concentrations. Calculated correlation coefficient: R = −0.693, significant at *p* < 0.001.

This effect was associated with a decrease in As root-to-shoot translocation factor TF along with increasing As root concentrations (Figure 4). Apparently, the mechanisms that prevented As from being translocated to the aboveground parts of plants were particularly efficient at high As root concentrations. 

Plant species-related values of the shoot BCF turned out to be the lowest (<0.1) for *A. capillaris*, known for its ability to develop various mechanisms of tolerance to high soil As [21,23,31], and the highest in the cases of *Dryopteris* spp. and *D. flexuosa* growing in acidic soils. Apparently, low soil pH and associated low extractability of As, did not prevent As from being taken up by plant roots and translocated to the shoots (fronds). This phenomenon should be more closely examined from the standpoint of plant physiology. Changing As uptake by plants in their various phonological phases should be considered as a factor of variability [75]. The median shoot BCF values, determined separately for each plant species, were in a relatively narrow range 3.4–12.4, with the highest values obtained for horsetail, ferns and red clover. A considerably efficient uptake of soluble As by horsetail and ferns can probably be explained by an intermittent increase in As solubility in periodically occurring wet conditions, typical for the habitats of area 6. Such an effect was not reflected by the results of As extractability, determined in dried soil samples. Seasonal differences in As accumulation by plants growing in floodplain soils and the importance of soil moisture conditions were clearly documented by Simmler et al. [39], similarly to the effects of soil moisture on As uptake by rice [8]. 

Similarly to the shoot BCFs, the root BCF values also showed a large diversity, although they were poorly correlated with each other (R = 0.273). It means that some plants that easily accumulated soluble As in their roots did not translocate it easily to the shoots. This observation reflects the differences between tolerant and non-tolerant plants. For instance, some samples of tolerant plant species: *L. corniculatus, H. lanatus, A. capillaris* and *F. rubra*, that had the root BCF values exceeding 1000, turned out to poorly translocate As to shoots. 

A lack of clear correspondence between As extractability with 1M NH_4_NO_3_ and As plant uptake, (in the whole data set and within the data characterizing single plant species), indicates that this extraction cannot be used for prediction of As accumulation by plants. Similar statement refers also to HNO_3_-extractable As. Consequently, a risk that As concentrations in plants growing in strongly enriched soils exceed a value of 4 mg/kg, considered a safe threshold, cannot be simply predicted based on soil extractions. 

Moreover, a multivariate PCA analysis (Figure S1), similarly to single correlations (Table 3) proved that shoot and root As concentrations correlate better with total than with extractable soil As. Though, the PCA analysis performed for single species separately indicated that (in some cases) the close associations between extractable As in soils and As in plants did exist, and those parameters were governed by the same principal components. For instance, the analysis indicated the strong associations between shoot and root As concentrations in major plant species, including *H. lanatus* and *C. epigejos* (Figure S1), which was reflected by their close neighborhood in related PCA graphs. In the case of ferns *Dryopteris* spp., however, there was no such an association, which means that different factors governed As accumulation in their roots (rhizomes) and fronds. 

Another kind of associations, revealed by the PCA graphs, was those among soil pH, “available” P, and (sometimes) a 1M NH_4_NO_3_-extractable soil As, which proved to be usually governed by the same principal components. It should be stressed again, however, that the concentrations of As in the shoots and roots of various plant species did not show any clear relationships with those soil parameters.

## 4. Conclusions

Our study showed that majority (over 66%) of plant samples collected from the sites affected by As mining and processing contained in their aboveground parts relatively high concentrations of As, above 4 mg/kg, an upper limit for safe animal fodder. Very high As concentrations in plant shoots were typical for extremely enriched soils, but they occurred also in the sites with total As below 1000 mg/kg. As concentrations in plant shoots proved to differ strongly among the species and within them. Despite the fact that they were correlated with total and extractable soil As, it would not be possible to simply predict As concentrations in plants based on soil parameters.

*Equisetum* spp. and *C. epigejos* indicated a particularly strong accumulation of As in their shoots, associated with the high values of TF. Very high shoot concentrations of As were also found in some samples of *A. capillaris*, in spite of the capability of this species to develop the mechanisms of tolerance that normally limit As root-to-shoot transfer. 

The cases of particularly intensive uptake of As by plants require a closer examination with special focus on possible seasonal and weather-related variations in soil As extractability and the dynamics of As accumulation by plants. Another issue that should be further investigated, is a pH-dependence of mechanisms responsible for As accumulation in plant roots and limiting its translocation to the aboveground parts of plants. Apparently, such mechanisms did not develop in plants growing in acidic mine soils.

## Figures and Tables

**Table 1:** Description of experimental areas and basic soil properties.

Mining Center	Area No	Description of Area, Settings	Soil Properties (Fine Soil) *			
			pH	As, mg/kg		
				Total	HNO_3_-Extract.	NH_4_NO_3_-Extract.
Złoty Stok (German: Reichenstein)	1	The Orchid Dump. A 2.4 ha large dump built of mine waste, covered ca. 50 years ago with a layer of humus-rich soil. It owes its name to a large population of *Orchis mascula* L. growing there [52,53]	3.14–5.81	751–48,900	42–10,500	0.08–4.88
	2	Hay meadows (ca. 6.0 ha) in a floodplain of the Trująca river, flooded periodically in the past by stormwater mixed with tailings [41]	3.53–6.66	102–6070	74–3650	0.30–6.04
	3	Foreland of tailings impoundment, a ca. 2 m-elevated plain area (1.6 ha) built of tailings [42]; dry, unmaintained grassland	7.22–7.60	7950–22,700	4710–9860	6.65–12.7
	4	Deep valley in a forested area, with spread dumps of mine wastes and heaps of slag disposed in medieval times by local smelting works [54]	3.43–4.89	1950–16,700	830–5090	1.45–1.52
Radzimowice (German: Altenberg)	5	Dumps of gangue rocks disposed at the Arnold shaft–A part of the Wilhelm mine that operated until 1925. Polymetallic veins of hydrothermal origin were exploited to acquire metals, mainly Fe, Cu, Pb and As [40]	2.90–7.26	1550–14,300	690–3320	0.20–1.56
	6	Forested area affected by acid mine and rock drainage (AMD and ARD) form Arnold shaft (Wilhelm mine) [55,56]	2.91–4.55	2480–193,000	650–18,900	0.04–27.5
	7	Hay meadows in the surroundings of two shafts of the Wilhelm mine. Soils contain admixtures of mine waste rocks. Additionally, they were polluted by the emissions from a local smelter that operated until 1925 [40]	3.60–4.39	73–603	5–78	0.02–0.67
Czarnow (German: Rothenzechau)	8	Dumps disposed by the Evelinensgluck mine that operated until 1925, and their close surroundings, partly forested, partly used as meadows and pastures [52,57,58]	2.88–7.43	72–98,500	4–6570	0.05–38.7

* All data are the mean values of three replicates, as explained in Section 2.2.

**Table 2:** Correlations coefficients between soil properties, total As and extractable As in soils, *n* = 146. The sets of data lacking a normal distribution were normalized by log-transformation.

As Parameter	Soil Parameters		
	Total As, mg/kg	pH	“Bioavailable” P
0.43M HNO_3_-extractable As, mg/kg	0.835 ***	0.316 ***	0.811 ***
1M NH_4_NO_3_-extractable As, mg/kg	0.726 ***	0.449 ***	0.786 ***
1M NH_4_NO_3_-extractable As, % of total	−0.445 ***	0.275 **	−0.057

Correlations significant at *p* < 0.01, and < 0.001 are indicated with asterisks (**, and ***, respectively)

**Table 3:** Correlations between soil properties and parameters that characterize As uptake by plants (*n* = 132). The sets of data lacking a normal distribution were normalized by log-transformation.

Parameter of As Uptake by Plants	Soil Parameters					
	Total As, mg/kg	0.43M HNO_3_-Extractable As, mg/kg	1M NH_4_NO_3_-Extractable As, mg/kg	pH	“Bio-Available” P, mg/kg	1M NH_4_NO_3_-Extractable As, % of Total
As in shoots, mg/kg	0.640 ***	0.584 ***	0.599 ***	0.430 ***	0.404 ***	−0.187 *
As in roots, mg/kg	0.645 ***	0.576 ***	0.503 ***	0.427 ***	0.485 ***	−0.227 **
TF	−0.274 **	−0.207 *	−0.122	−0.116	−0.167 *	0.223 **
BAF–shoots	−0.674 ***	−0.549 ***	−0.379 **	−0.133	−0.338 **	0.368 ***
BAF–roots	−0.361 ***	−0.287 **	−0.223 **	−0.046	−0.188 *	0.193
BCF–shoots	−0.277 **	−0.481 **	−0.641 ***	−0.282 **	−0.473 ***	−0.471 ***
BCF–roots	−0.016	−0.202 **	−0.531 ***	−0.101	−0.234 **	−0.531 ***

Correlations significant at *p* < 0.05, 0.01, and 0.001 are indicated with asterisks (*, **, and ***, respectively).

**Table 4:** Arsenic BAF and BCF values calculated for shoots and roots of particular plant species. BCF related to 1M NH_4_NO_3_-extractable As in soils.

Species	Shoot BAF		Shoot BCF		Root BAF		Root BCF	
	Range	Median	Range	Median	Range	Median	Range	Median
*Acer platanoides*	<0.001–0.014	0.001	1.3–103	3.4	0.001–0.131	0.020	7.2–410	45
*Picea abies*	<0.001–0.006	<0.001	0.4–8.3	4.5	<0.002–0.018	0.004	1.1–32.8	19
*Holcus lanatus*	<0.001–0.066	0.005	0.7–206	7.9	0.002–0.624	**0.051**	3.7–2910	**78**
*Festuca rubra*	<0.001–0.174	0.003	<0.1–284	4.9	<0.001–0.355	0.018	15.4–217	**106**
*Agrostis capillaris*	<0.001–0.116	0.005	<0.1–74	6.4	<0.001–0.885	**0.054**	<0.1–8290	49
*Deschampsia flexuosa*	<0.001–0.015	0.001	1.3–1120	6.3	<0.001–0.025	0.002	0.5–1350	10
*Calamagrostis epigejos*	0.002–0.179	0.005	0.9–204	**10.0**	0.006–1.13	**0.062**	7.9–2790	49
*Calamagrostis arundin.*	0.001–0.001	0.001	0.8–9.7	5.9	0.004–0.052	0.009	22.7–114	46
*Lotus corniculatus*	<0.001–0.164	0.005	1.4–51	4.5	0.001–0.164	**0.059**	7.3–1360	14
*Trifolium pretense*	0.001–0.062	0.004	0.8–265	**12.4**	0.021–0.065	0.033	15.4–217	**107**
*Silene vulgaris*	0.001–0.005	0.002	1.3–12	5.4	0.004–0.063	0.018	6.3–170	29
*Dryopteris spp.*	<0.001–0.105	0.002	2.7–1210	**10.8**	<0.001–0.069	0.003	2.9–283	22
*Equisetum spp.*	<0.001–0.047	**0.008**	1.2–96.6	**15.3**	0.001–0.135	0.019	0.7–347	**79**

Particularly high median values are highlighted in bold.

## Supplementary Materials

The following are available online at https://www.mdpi.com/1660-4601/17/9/3342/s1, Table S1: Plant species examined; Table S2: Median As concentrations in shoots and roots and median values of transfer factor TF for plant species examined; Figure S1: The results of principal component analysis performed for all data and selected plant species separately.
