# Supplementary material for: Accumulation of Arsenic by Plants Growing in the Sites Strongly Contaminated by Historical Mining in the Sudetes Region of Poland
Source: Int J Environ Res Public Health. 2020 May 11;17(9):3342. doi: 10.3390/ijerph17093342 (PMC7246468; doi:10.3390/ijerph17093342)
Supplement: Supplementary file 1 [file ijerph-17-03342-s001.pdf]

International Journal of Environmental Research and Public Health

MDPI

Table S1. Plant species examined.

Plant Group	Species	No of Samples	Sites
Trees (seedlings)	*Acer platanoides* L. (seedlings)	9	1, 4, 6, 8
	Picea abies L. (seedlings)	4	4, 5, 6, 8
Grasses	*Holcus lanatus* L.	26	1, 2, 5, 6, 7, 8
	Festuca rubra L.	29	1, 2, 3, 5, 7, 8
	Agrostis capillaris L.	29	1, 2, 5, 6, 7, 8
	Deschampsia flexuosa L.	9	1, 5, 6, 8
	Calamagrostis epigejos L.	11	1, 2, 3
	Calamagrostis arundinacea L.	4	4
Fabaceae	*Lotus corniculatus* L.	11	1, 2, 3, 5, 7, 8
	Trifolium pretense L.	4	1, 2, 7
Other plants	*Silene vulgaris* L.	5	1, 7, 8
	Dryopteris spp. L.	15	1, 4, 5, 6, 8
	Equisetum spp. L.	12	2, 6, 7, 8

Table S2. Median As concentrations in shoots and roots and median values of transfer factor TF for plant species examined.

Plant Group	Species	Median As Concentrations in Plant Material, mg/kg		Median TF Values
		Shoots	Roots	
Trees (seedlings)	*Acer platanoides* L. (seedlings)	5.8	48.8	0.11
	Picea abies L. (seedlings)	8.3	38.9	0.37
Grasses	*Holcus lanatus* L.	8.3	44.5	0.19
	Festuca rubra L.	5.5	45.8	0.15
	Agrostis capillaris L.	5.6	23.5	0.17
	Deschampsia flexuosa L.	7.5	11.3	0.82
	Calamagrostis epigejos L.	26.8	146.8	0.14
	Calamagrostis arundinacea L.	8.9	69.6	0.13
Fabaceae	*Lotus corniculatus* L.	15.8	10.8	0.17
	Trifolium pretense L.	3.9	35.8	0.15
Other plants	*Silene vulgaris* L.	9.8	125.8	0.16
	Dryopteris spp. L.	12.8	32.3	0.21
	Equisetum spp. L.	47.5	15.3	0.28

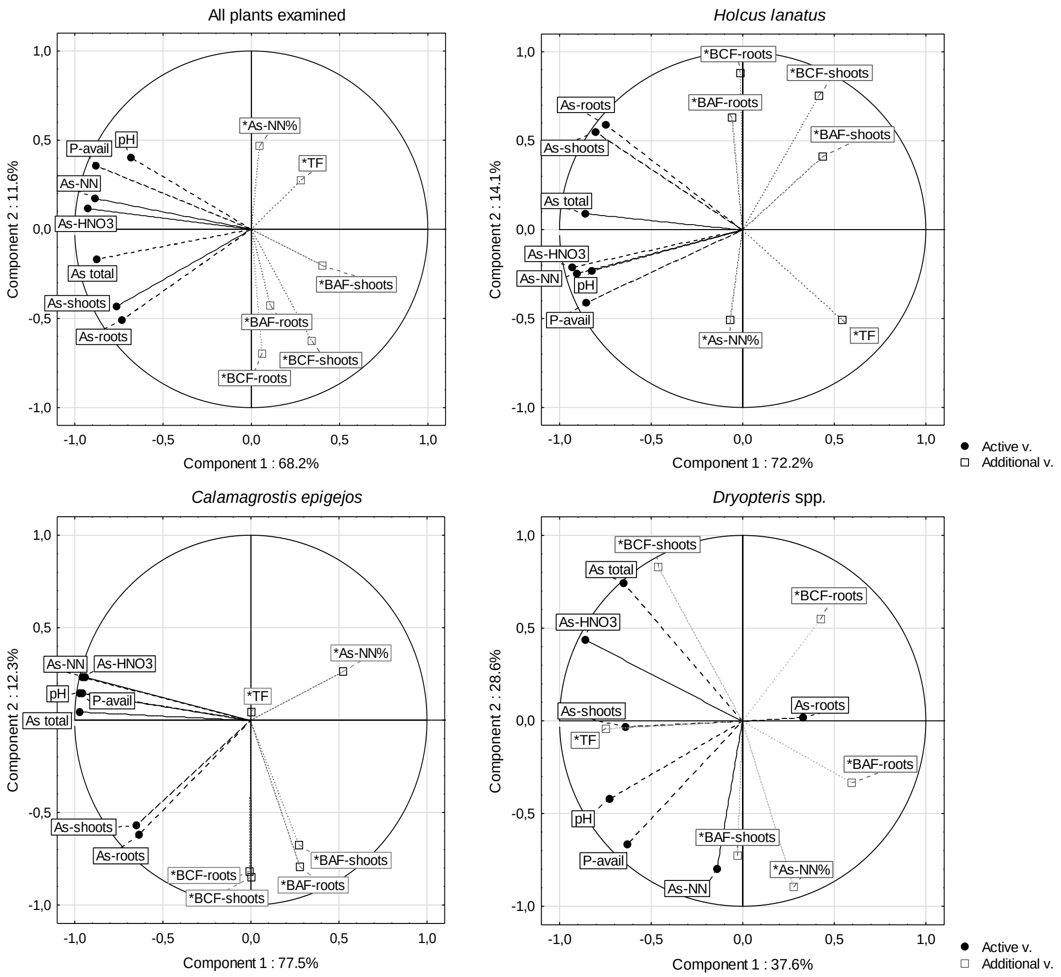

Figure S1. The results of principal component analysis performed for all data and selected plant species separately. The graphs illustrate relationships between the main variables that characterize soil properties and As uptake by plants-as related to principal Components 1 and 2. Active values in PCA analysis have been indicated with black dots. Plant species separately selected for PCA analysis: *Calamagrostis epigejos, Holcus lanatus,* and *Dryopteris* spp. Explanations: As-HNO3: soil concentrations of 0.43M HNO_3-extractable As, As-NN: soil concentrations of 1M NH_4NO_3-extractable As, As-NN%: shares of 1M NH_4NO_3-extractable As in total As (expressed in %), P-avail–extractable P in soil, BCF-shoots and BCF-roots: BCF factors related to 1M NH_4NO_3-extractable As.
